# Physical frailty predicts the development of social frailty: a prospective cohort study

**DOI:** 10.1186/s12877-020-01814-2

**Published:** 2020-10-14

**Authors:** Koutatsu Nagai, Kayoko Tamaki, Hiroshi Kusunoki, Yosuke Wada, Shotaro Tsuji, Masako Itoh, Kyoko Sano, Manabu Amano, Seiya Hayashitani, Ryota Yokoyama, Ryo Yonezawa, Tsukasa Kamitani, Ken Shinmura

**Affiliations:** 1 Department of Physical Therapy, School of Rehabilitation, Hyogo University of Health Sciences, 1-3-6 Minatojima, Chuo-ku, Kobe, Hyogo 650-8530 Japan; 2 Department of General Medicine, Hyogo College of Medicine, 1-1 Mukogawa, Nishinomiya, Hyogo 663-8501 Japan; 3 Department of Rehabilitation, Hyogo College of Medicine Sasayama Medical Center, 5 Kurooka, Sasayama, Hyogo 669-2321 Japan; 4 Department of Orthopaedic Surgery, Hyogo College of Medicine, 1-1 Mukogawa, Nishinomiya, Hyogo 663-8501 Japan; 5 Department of Occupational Therapy, School of Rehabilitation, Hyogo University of Health Sciences, 1-3-6 Minatojima, Chuo-ku, Kobe, Hyogo 650-8530 Japan; 6 School of Pharmacy, Hyogo University of Health Sciences, 1-3-6 Minatojima, Chuo-ku, Kobe, Hyogo 650-8530 Japan; 7 Department of Healthcare Epidemiology, School of Public Health in the Graduate School of Medicine, Kyoto University, Yoshidakonoe-cho, Sakyo-ku, Kyoto, Kyoto 606-8501 Japan

**Keywords:** Social frailty, Physical frailty, Older adults

## Abstract

**Background:**

It has not been clarified whether physical frailty symptoms predict social.

frailty. The purpose of this study was to elucidate the effect of physical frailty on social frailty, and to determine which domains of physical frailty predict the development of social frailty.

**Methods:**

We employed a two-year prospective cohort study. A total of 342 socially robust community-dwelling older adults were recruited. We used a modified social frailty screening index consisting of four social domains including financial difficulties, living alone, social activity, and contact with neighbors. Physical frailty status was also assessed at baseline. At the two-year follow-up, we assessed the development of social frailty. Social status was assessed using four social subdomains for the primary analysis. Social status was assessed using the two social subdomains of social activity and contact with neighbors, which would be affected by the physical frailty component, for the secondary analysis. The risk ratios (RR) of physical frailty for the development of social frailty were estimated.

**Results:**

Although physical frailty symptoms were not a significant risk factor for future development of social frailty as assessed by four social subdomains (adjusted RR 1.39, 95% CI 0.95–2.15), it became significant when development of social frailty was assessed by the two social subdomains (adjusted RR 1.78, 95% CI 1.10–2.88). An analysis using the physical frailty subdomain showed that slow gait speed (adjusted RR 3.41, 95% CI 1.10–10.53) and weakness (adjusted RR 1.06, 95% CI 1.01–1.12) were independent risk factors for development of social frailty as assessed by two social subdomains.

**Conclusions:**

Physical frailty symptoms predict the development of social frailty. Among physical frailty subdomains, gait speed and muscle strength are critical independent risk factors for future decline in the social aspect. The prevention of physical frailty, especially by maintaining gait ability and muscle strength, may be effective for avoiding social frailty.

## Background

Frailty is a state of vulnerability that results in an increased risk of adverse health outcomes related to aging [1]. The concept of frailty includes physical, psychological, and social components [2]. Among them, physical frailty is the most commonly investigated component and has been shown in many studies to predict disability, hospitalization, and mortality [1, 3, 4].

Recently, social frailty has also attracted attention. Social frailty in aging populations is of grave concern because of social issues faced by the elderly such as those related to income, family dynamics, and social inclusion [5]. Although a gold standard assessment for social frailty has not been established yet, several studies have demonstrated the adverse health outcomes attributable to social frailty, such as disability and mortality [5–7]. Thus, the prevention of social frailty constitutes an important issue in an ageing society.

Social frailty is one of the risk factors for physical frailty [8], indicating that there is a link between social and physical frailty. Although the basis for the causal relationship between the social frailty component and physical frailty has not necessarily been clarified well, some researchers ascertained that limited social activity or connection, which is a components of social frailty, led to the decline of physical activity (PA) and functioning [9–12].

Meanwhile, this temporal relationship, namely that social frailty leads to physical frailty, may be a reciprocal rather than a one-way relationship. An earlier study reported that physical functional decline caused limited life-space mobility [13], which would restrict outdoor social activity. However, it has not been clarified whether physical frailty symptoms induce social frailty. We here hypothesize that physical frailty symptoms are a risk factor for social frailty. Further, it is unclear which subdomains of physical frailty closely predict the decline of the social aspect. This knowledge would aid the development of an intervention strategy to prevent a downward spiral of frailty. The purpose of this study was to elucidate the effect of physical frailty on the social frailty aspect, and to determine which domains of physical frailty strongly predict the decline of the social aspect.

## Methods

### Study design and participants

This study uses a prospective cohort analysis design. Baseline information was gathered in the period September 2015 to December 2017 from the study on Frail Elderly in the Sasayama-Tamba Area (FESTA). Tamba-Sasayama City, with a population of 41,490 as of 2015, is situated in the suburban area of the Hyogo prefecture. The average age of the city’s population is higher than the Japanese average (31.4% aged 65 or above). We recruited community-dwelling older adults to participate by using an community advertisement, placing posters at Sasayama Medical Center, and oral announcements by medical staff in the city. The two-year follow-up assessment was conducted between September 2017 and December 2019 to assess the incidence of the decline of the social frailty component.

The present study targeted participants who at least engaged in social activity or had contact with neighbors, which are potentially related to physical frailty, among subdomains of social frailty. The inclusion criteria were: (1) aged 65 years or older, (2) able to walk independently with/without a cane, and (3) not socially frail/pre-frail at baseline. The exclusion criteria were: (1) cognitive impairment, which was determined by a Mini-Mental State Examination (MMSE) score less than 21 [14], and (2) participants containing missing data.

During the initial phase, 625 older adults participated in the baseline assessment. Among them, 376 met the inclusion criteria (out of 625, 201 were pre-socially frail, and 48 were socially frail). One person was excluded due to cognitive impairment. Out of 375 people, 352 participated in the secondary assessment (23 dropped out), and 10 people had missing data. Accordingly, the study comprised the remaining 342 participants.

### Social frailty

We used a modified social frailty index [5] founded on Bunt’s social frailty concept, which measures general and social resources, social behaviors, and the satisfaction of basic social requirements [15]. The screening index was developed to briefly assess social frailty status, which can predict future incidents of activity limitation and mortality in community-dwelling older adults [5]. The question regarding general resources (financial difficulties) is: “Do you have a financial problem in your daily life?” Financial difficulty was defined as a “yes” answer. The question regarding social resources (living alone) is: “How many people do you live with?” Inadequate social resources were reflected in the answer “alone.” The question pertaining to social behavior (lack of social activity) is: “Do you participate in any community activities or volunteer activities?” Poor social behavior was defined as an answer “none.” The question assessing the satisfaction of basic social requirements (lack of contact with neighbors) is: “Do you sometimes visit your friends?” Deficiency in basic social needs was identified as a “no” answer. A score of 2 or more was defined as social frailty, 1 as pre-social frailty, and 0 as social robustness.

### Physical frailty

We assessed physical frailty status according to the Fried phenotype [1, 16]: (1) slow gait speed, (2) weakness, (3) exhaustion, (4) low activity, and (5) weight loss. Participants who did not show any of these five symptoms were considered non-frail, while those with one or two symptoms were defined as pre-frail. For the assessment of gait speed, we asked participants to cover a 12 m walkway at their usual speed. Then, the time for 10 m in the way was assessed [17]. Slowness was determined by a cut-off point less than 1.0 m/s [1]. We measured maximum grip strength by using a grip strength tester (GRIP-A; Takei Ltd., Niigata, Japan). Strength weakness was identified according to established cut-off (< 26 kg for men, < 18 kg for women) [18]. We assessed exhaustion with the following question from the Kihon Check List [19]: “In the last two weeks, have you felt tired without a reason?” Weight loss was assessed with the following question: “Have you lost 2 kg or more in the past six months?” [19]. PA was assessed with a wrist wearable accelerometer (Actiband, TDK Co., Tokyo, Japan) for 14 days. The epoch duration for recording PA was 5 min. The reliability and validity of this accelerometer have been confirmed previously [20]. Data from participants with complete measurements recorded for at least 3 days were included in the analysis. Participants who did not record at least 600 min of PA were excluded [21]. We defined lower than 1 standard deviation away from mean of moderate to vigorous PA (MVPA, ≥3 METs) [22] in the participants as low PA.

### Other variables

Each subject’s age, sex, comorbidity, and education were self-reported. We used the Geriatric Depression Scale (GDS) [23] to assess depressive symptoms. We also assessed the Instrumental Activity of Daily Living (IADL) using the Tokyo Metropolitan Institute of Gerontology Index of Competence (TMIG-IC) [24] and participants’ history of falls in the previous year.

### Outcome measure

The social frailty score (four domains) was used as the primary outcome. We re-assessed it during the two-year follow-up, and categorized participants into a socially maintained or socially declined group according to their change in social score (four domains) to clarify the temporal relationship between physical frailty and social frailty. As the secondary analysis, we focused on social behavior (social activity) and fulfillment of basic social needs (contact with neighbors) in the social frailty index, which would be affected by the physical components because the two activities require physical movement. We accordingly excluded the remaining two social variables in the secondary analysis, general resources (economic hardship), and social resources (living alone), because they may be difficult to control using the physical aspect in older adults.

### Statistical analysis

The participant’s characteristics assigned to the physically robust and pre-frailty or frailty (with any frailty subdomain) groups at the baseline were compared by using a Student’s t-test, Mann–Whitney U, or chi-square tests according to the type of variables. We also assessed the baseline differences between the socially maintained and socially declined groups (four domains) during the two-year follow-up. A modified Poisson regression model [25] was used to estimate the risk ratio (RR) and 95% confidence intervals (CIs) of physical frailty for the development of social frailty (four domains) in the crude and adjusted model as a primary analysis. First, the physical frailty condition (robust or any-frailty) was included as an independent variable. Second, five subdomains of physical frailty were entered using the forced entry method. Of those, we added walking speed, handgrip strength, and PA as continuous variables. The adjusted model added age, sex, MMSE, GDS, multimorbidity (two or more chronic illnesses), IADL score, and fall experience as covariates. As a secondary analysis, we assessed the incidence risk of development of social frailty focusing on social activity and contact with neighbors in the social frailty index, domains which possibly have a direct relationship with physical components. Data were analyzed using IBM SPSS ver. 24 (IBM Japan Ltd., Tokyo, Japan). Statistical significance was set at *p* < 0.05.

## Results

Table 1 presents the participants’ characteristics according to their physical frailty condition at baseline. Of the 342 participants, 232 (68%) were classified as physically robust and 110 (32%) showed one or more physical frailty symptoms. Participants classified as physically any level of frailty showed a higher GDS score, higher prevalence of history of falls, lower gait speed, lower strength, and lower MVPA.

**Table 1:** Baseline characteristics in participants

Variables	Overall (*n* = 342)	Physically Robust (*n* = 232)	Physically any-frail (*n* = 110)	*P*-value
Age, y, mean (SD)	72.6 (5.6)	72.2 (5.1)	73.4 (6.3)	0.079
Women, n (%)	217 (64)	146 (63)	71 (65)	0.772
Height, cm, mean (SD)	156.4 (8.2)	156.6 (8.2)	156.0 (8.4)	0.488
Body mass index, kg/m^2^, mean (SD)	22.6 (3.0)	22.4 (2.8)	23.0 (3.3)	0.090
Medication, n, median (IQR)	1 (0–3)	1 (0–2)	1 (0–3)	0.088
Comorbidities				
Hypertension, n (%)	157 (46)	101 (44)	56 (51)	0.201
Diabetes, n (%)	39 (11)	28 (12)	11 (10)	0.472
Kidney disease, n (%)	13 (4)	7 (3)	6 (6)	0.363
Cardiovascular disease, n (%)	27 (8)	19 (8)	8 (7)	0.769
Osteoporosis, n (%)	41 (12)	24 (10)	17 (16)	0.174
Multimorbidity, n (%)	103 (30)	66 (28)	37 (34)	0.329
MMSE, median (IQR)	29 (27–30)	29 (27–30)	29 (27–30)	0.273
Education, y, median (IQR)	12 (12–14)	12 (12–14)	12 (12–14)	0.916
GDS, median (IQR)	0 (0–2)	0 (0–0)	1.5 (0–3)	< 0.001
History of falls, n (%)	76 (22)	39 (17)	37 (34)	< 0.001
TMIG-IC, median (IQR)	13 (13–13)	13 (13–13)	13 (13–13)	0.318
Gait speed, m/s (SD)	1.5 (0.2)	1.6 (0.2)	1.4 (0.2)	< 0.001
Handgrip strength, kg (SD)	28.6 (7.6)	29.2 (7.5)	27.5 (7.7)	0.065
MVPA, METs・hour (SD)	3.2 (2.5)	3.6 (2.6)	2.3 (2.0)	< 0.001
Subdomain of physical frailty				
Slow gait speed, n (%)	6 (2)	–	6 (5)	–
Weakness, n (%)	12 (4)	–	12 (11)	–
Exhaustion, n (%)	55 (16)	–	55 (50)	–
Low activity, n (%)	24 (7)	–	24 (22)	–
Weight loss, n (%)	41 (12)	–	41 (37)	–

*SD* Standard deviation, *IQR* Interquartile range, *GDS* Geriatric depression scale, *TMIG-IC* Tokyo Metropolitan Institute of Gerontology Index of Competence, *MVPA* Moderate to vigorous physical activity

χ2 test or Fisher exact test for proportions, Student t-test for parametric variables and Mann–Whitney U test for non-parametric variables

At the two-year follow-up, 12 (4%) of 342 participants were categorized as socially frail, and 53 (15%) were socially pre-frail (Table 2); overall, 65 participants showed some development of social frailty. The social changes in each subdomain are shown in Table 2. Eleven people (3%) began to feel financial difficulty, and eleven people (3%) changed to living alone. Regarding lack of social activity, 50 participants (15%) lost their activity. Six people (2%) lost their ability to have contact with neighbors. By comparing baseline characteristics between the socially maintained and socially declined groups (by four domains) during the two-year follow-up, there were significant differences in age, sex, height, GDS, history of falls, gait speed, handgrip strength, and MVPA.

**Table 2:** Baseline differences between the socially robust and socially declined groups during the two-year follow-up

Variables	Socially Robust (*n* = 277)	Socially declined (*n* = 65)	*P*-value
Age, y, mean (SD)	72.0 (5.3)	75.0 (6.3)	< 0.001
Women, n (%)	167 (60)	50 (77)	0.012
Height, cm, mean (SD)	157.2 (8.2)	153.2 (7.7)	< 0.001
Body mass index, kg/m^2^, mean (SD)	22.7 (3.0)	22.2 (3.0)	0.292
Medication, n, median (IQR)	1 (0–3)	2 (0–3)	0.599
Multimorbidity, n (%)	66 (28)	37 (34)	0.184
MMSE, median (IQR)	29 (27–30)	28 (26–30)	0.081
Education, y, median (IQR)	12 (12–14)	12 (12–14)	0.933
GDS, median (IQR)	0 (0–1)	0 (0–2)	0.035
History of falls, n (%)	39 (17)	37 (34)	0.030
TMIG-IC, median (IQR)	13 (13–13)	13 (13–13)	0.723
Gait speed, m/s (SD)	1.5 (0.2)	1.4 (0.2)	0.006
Handgrip strength, kg (SD)	29.3 (7.8)	25.6 (6.1)	< 0.001
MVPA, METs・hour (SD)	3.4 (2.6)	2.5 (1.9)	0.012
Physically any-frailty	83 (30)	27 (42)	0.072
Subdomain of physical frailty			
Slow gait speed, n (%)	4 (1)	2 (3)	0.320
Weakness, n (%)	8 (3)	4 (6)	0.198
Exhaustion, n (%)	42 (15)	13 (20)	0.339
Low activity, n (%)	16 (6)	8 (12)	0.064
Weight loss, n (%)	33 (12)	8 (12)	0.930
Socially Robust, n (%)	277 (100)	–	–
Socially pre-frail, n (%)	–	53 (82)	–
Socially frail, n (%)	–	12 (18)	–
Subdomain of social frailty			
Financial difficulty, n (%)	–	11 (3)	–
Living alone, n (%)	–	11 (3)	–
Lack of social activity, n (%)	–	50 (15)	–
Lack of contact with neighbors, n (%)	–	6 (2)	–

*SD* Standard deviation, *IQR* Interquartile range, *GDS* Geriatric depression scale, *TMIG-IC* Tokyo Metropolitan Institute of Gerontology Index of Competence, *MVPA* Moderate to vigorous physical activity

χ2 test or Fisher exact test for proportions, Student t-test for parametric variables and Mann–Whitney U test for non-parametric variables

According to the modified Poisson regression, the presence of physical frailty symptoms was not a significant risk factor for development of social frailty by four domains (crude RR 1.50, 95% CI 0.97–2.32, adjusted RR 1.39, 95% CI 0.90–2.15) (Table 3). An analysis using the physical frailty subdomain showed that weakness was an independent risk factor for development of social frailty by four subdomains (adjusted RR 1.05, 95% CI 1.00–1.11). Meanwhile, focusing on development of social frailty by two domains (social activity and contact with neighbors), physical frailty symptoms were significantly related to development of social frailty. Subdomain analysis showed that slow gait speed and muscle weakness were significant risk factors for development of social frailty by two domains.

**Table 3:** Risk ratios for the development of social frailty after two years according to physical frailty subdomains

	Crude model			Adjusted model		
	RR	95%CI	*p* value	RR	95%CI	*p* value
Social frailty (by 4 domains)						
Physical frailty symptom	1.50	0.97–2.32	0.070	1.39	0.90–2.15	0.134
Subdomain						
Slow gait speed	2.70	1.05–6.94	0.040	2.65	0.94–7.52	0.066
Weakness	1.06	1.02–1.09	0.003	1.05	1.00–1.11	0.045
Exhaustion	1.07	0.63–1.83	0.799	0.99	0.58–1.71	0.979
Low activity	1.09	0.97–1.22	0.162	1.08	0.96–1.21	0.214
Weight loss	0.90	0.46–1.77	0.760	0.92	0.47–1.81	0.816
Social frailty (by 2 domains)						
Physical frailty symptom	1.88	1.15–3.07	0.011	1.78	1.10–2.88	0.020
Subdomain						
Slow gait speed	3.15	1.07–9.35	0.037	3.41	1.10–10.53	0.033
Weakness	1.06	1.02–1.10	0.005	1.06	1.01–1.12	0.031
Exhaustion	1.23	0.68–2.21	0.490	1.14	0.64–2.04	0.655
Low activity	1.10	0.95–1.27	0.197	1.10	0.96–1.26	0.189
Weight loss	0.94	0.43–2.03	0.869	0.94	0.43–2.05	0.880

All of the subdomains in the social frailty index were used to assess development of social frailty (by 4 items). Social frailty (by 2 items) was assessed by the social activity and contact with neighbors subdomains in the social frailty index

Slow gait speed, weakness (handgrip strength), and low activity levels were added as continuous variables in the models. Their RRs represent the risk when gait speed, handgrip strength, and physical activity are reduced by 1.0 m/s, 1.0 kg, and 1.0 METs・hour, respectively

The adjusted model was adjusted with age, sex, GDS, MMSE, multimorbidity, fall, and TMIG-IC (IADL)

*RR* Risk Ratio

## Discussion

The current prospective cohort study investigated the effect of physical frailty on the social frailty, and assessed the effect of each subdomain of physical frailty on the future decline of the social aspect. Physical frailty symptoms were not necessarily related to the development of social aspects assessed by all subdomains. Only muscle weakness was associated with development of social frailty. Meanwhile, when focusing on social aspects assessed by the two subdomains of social activity and contact with neighbors, physical frailty symptoms were a risk factor predictive of development of social frailty. Among the subdomains of physical frailty, slow gait speed and muscle weakness were the critical domains that related to changes in them.

The effects of the social aspect on physical condition have been reported in several studies. Earlier studies have uncovered that the decline of social aspects in older people, including social relations, social engagement, and social connection, was related to physical functioning decline [10, 26, 27]. In recent studies, social frailty emerged as a risk factor for the incidence of physical frailty, disability and mortality [5–8]. Poor social connectedness contributes to low PA [10], which likely induces a decline of physical functioning [12]. On the contrary, the present study demonstrated a reverse relationship in that physical frailty leads to social aspect declines assessed by social activity and contact with neighbors. Older adults with physical limitations are likely to be physically inactive [11], and low physical functioning restricts life-space mobility [13], which likely restricts outdoor social activity. Considering the relationship between physical and social factors reported previously, the results of the present study seem to be reasonable.

The significant relationship between physical and social frailty was underlined when focusing on the two social domains of social activity and contact with neighbors, rather than on the four social domains that include living alone and financial difficulty. A change to living alone seems to be an environmental factor, which is difficult to control through personal physical function because it includes death in the family. Regarding financial difficulty, recruited participants were aged 65 or older in this study; thus, it is considered that many people are already retired and living on pension in Japan. Financial conditions in those people may not be affected by physical aspects. Based on these ideas, we presumed that the relationship between physical frailty and social frailty would become obvious in the secondary analysis. Out of the four domains, the development of lack of social activity was the most prevalent (15%). We assume that outdoor social activity requires higher physical function than contact with neighbors, which can be achieved in the limited area around their house. Assessing lack of social activity may thus lead to better sensitivity for assessing the development of social frailty.

Among the physical frailty subdomains, lower gait speed and muscle strength were independently predictive of development of social frailty. An earlier study reported that low physical functioning was a risk factor for limited life-space mobility [13]. The ability to walk safely is a prerequisite to going outdoors independently [28]. We therefore consider the present result of slow gait speed as a relevant risk factor. Regarding the relationship between the social aspect and muscle weakness, Haraldstad et al. conducted the intervention with strength training and reported that increase of strength was related to improvement in social function [29]. The other study mentioned a negative association with social function (including social contact with friends and neighbors) and fear of falling in the elderly [30]. The fear of falling is a known risk factor for avoidance of going outside [31], and this fear has been reported to be related to muscle weakness [32]. Accordingly, muscle weakness may be a predictor of future social frailty via fear of falling. The results of this study suggest the possibility that intervention that targets gait speed and muscle strength is effective in avoiding future social frailty among older adults.

Our results coupled with the reported result that social frailty predicts physical frailty [8], implicate a hypothetical model wherein social frailty and physical frailty are interinfluenced. The model could provide a new intervention concept that will help prevent the negative loop of social/physical frailty. However, the present study was an observational study, which potentially include bias and unknown confounders; thus, causal associations between physical and social aspects should be carefully interpreted. Additionally, the relationship between social and physical aspects are not necessarily simple. Ma et al. demonstrated that social frailty was associated with not only physical functioning, but also cognitive function and depression [7]. Clinicians may need to consider the interactions of psychological and physical functioning in order to prevent social frailty in the elderly. Leastwise, present results suggest the necessity of supposing reciprocal effects between physical and social factors, as well as the importance of assessing both factors to understand the future risk of adverse health outcomes.

The present study had some limitations. First, participants were recruited via an advertisement in a community newspaper or oral announcements, depending on their motivation to participate. This may have involved a bias in that the recruited participants tended to have lower development of social frailty. Second, we did not include participants with cognitive decline to prioritize the reliability of assessments. Cognitive impairment would influence social aspects [7]. This study could not make reference to the effect of cognitive impairment on the relationship between physical and social frailty. Third, we could not consider the effect of geographical information and trauma on the development of social frailty.

## Conclusions

Our study underscores the fact that symptoms of physical frailty often predict the development of social frailty. Additionally, among the subdomains of physical frailty, lower gait speed and muscle strength are critical independent risk factors for future decline of social aspects. The prevention of physical frailty, especially by maintaining gait ability and muscle strength, may be an effective solution for avoiding social frailty.

## Data Availability

The datasets used and/or analyzed during the current study are available from the corresponding author on reasonable request.

## Abbreviations

RR: Risk Ratio
PA: Physical Activity
MMSE: Mini-Mental State Examination
MVPA: Moderate to Vigorous Physical Activity
IADL: Instrumental Activity of Daily Living
TMIG-IC: Tokyo Metropolitan Institute of Gerontology Index of Competence
GDS: Geriatric Depression Scale
CI: Confidence Interval
